# First‐generation HapMap in *Cajanus* spp. reveals untapped variations in parental lines of mapping populations

**DOI:** 10.1111/pbi.12528

**Published:** 2016-01-29

**Authors:** Vinay Kumar, Aamir W. Khan, Rachit K. Saxena, Vanika Garg, Rajeev K. Varshney

**Affiliations:** ^1^ International Crops Research Institute for the Semi‐Arid Tropics (ICRISAT) Hyderabad India; ^2^ School of Plant Biology and Institute of Agriculture The University of Western Australia Crawley WA Australia

**Keywords:** deletions, insertions, next‐generation sequencing, pigeonpea, single‐nucleotide polymorphism, whole genome re‐sequencing

## Abstract

Whole genome re‐sequencing (WGRS) was conducted on a panel of 20 *Cajanus* spp. accessions (crossing parentals of recombinant inbred lines, introgression lines, multiparent advanced generation intercross and nested association mapping population) comprising of two wild species and 18 cultivated species accessions. A total of 791.77 million paired‐end reads were generated with an effective mapping depth of ~12X per accession. Analysis of WGRS data provided 5 465 676 genome‐wide variations including 4 686 422 SNPs and 779 254 InDels across the accessions. Large structural variations in the form of copy number variations (2598) and presence and absence variations (970) were also identified. Additionally, 2 630 904 accession‐specific variations comprising of 2 278 571 SNPs (86.6%), 166 243 deletions (6.3%) and 186 090 insertions (7.1%) were also reported. Identified polymorphic sites in this study provide the first‐generation HapMap in *Cajanus* spp. which will be useful in mapping the genomic regions responsible for important traits.

## Introduction

Pigeonpea (*Cajanus cajan* L.) belongs to the genus *Cajanus* under *Fabaceae* family. The genus *Cajanus* is comprised of 32 species including wild and cultivated species. Pigeonpea is one of the most important food legume crops grown in marginal environments of the world. It has been considered a rich source of protein to the vegetarian families. However, the crop productivity has remained stagnant (~750 kg/ha) during last six decades (http://faostat.fao.org/). The low level of yield is due to biotic stresses [fusarium wilt (FW), sterility mosaic disease (SMD), etc.], abiotic stresses (water logging, salinity, etc.) and narrow genetic base in the cultivated gene pool (Saxena *et al*., 2010). Research efforts have been undertaken to solve the above‐mentioned constraints and a number of disease‐resistant and high‐yielding varieties were released for cultivation. However, the average yield level remains <1 ton per hectare (http://www.iipr.res.in/aicrp.html).

Genomics‐assisted breeding (GAB) has been successfully deployed in a number of crops species to tackle the long‐standing problems (Varshney *et al*., 2009). In the case of pigeonpea, GAB could not be adequately deployed to tackle the constraints responsible for low yield. In terms of genomics resources, large number of molecular markers such as simple sequence repeat (SSR), diversity array technology (DArT), single‐feature polymorphism (SFP), single‐nucleotide polymorphisms (SNPs), etc., and a number of segregating populations have been developed (Bohra *et al*., 2014; Dutta *et al*., 2011; Pazhamala *et al*., 2015). Mapping for economical important traits could not be very successful with above‐mentioned resources as only few hundreds of markers were found polymorphic in parental genotypes (Bohra *et al*., 2014).

Next‐generation sequencing (NGS) technologies have provided new avenues to detect genome‐wide variations present in gene pools of different species (Bevan and Uauy, 2013; Thudi *et al*., 2012). After having a draft genome in a particular crop species, deploying NGS becomes more cost effective in assessing the genome‐wide variations (Varshney *et al*., 2009). NGS enables identification of SNPs and InDels in efficient and high‐throughput manner (Lam *et al*., 2010; Xu *et al*., 2012). At present, draft genome sequences have become available in a number of crop species (Michael and Jackson, 2013) including pigeonpea (Singh *et al*., 2011; Varshney *et al*., 2012). Further, in the recent past, NGS has been used in identifying genome‐wide variations through WGRS based approaches (Arai‐Kichise *et al*., 2014; Mace *et al*., 2013; Varshney *et al*., 2009; Zheng *et al*., 2011). This will subsequently enable the discovery of SNPs and InDels at the genome‐wide scale within both germplasm collections and breeding lines of pigeonpea. SNPs and InDels are of high importance for crop improvement programs (Chen *et al*., 2014; Tao *et al*., 2014). Both SNPs and InDels are being used in different trait mapping approaches, haplotype analysis and GAB programs such as marker‐assisted selection, marker‐assisted back‐crossing, genomic selection, varietal identification and hybridity testing. In recent years, SNPs have been detected and analysed in many cultivars and inbred lines of pigeonpea and used for linkage mapping and diversity analysis (Saxena *et al*., 2012, 2014). However, these SNPs were identified in limited number of accessions and few SNPs were applicable to other accessions. For implementing modern GAB methodologies, it is highly imperative to detect genome‐wide variations in a larger set of accessions.

In view of above, we have re‐sequenced 20 diverse *Cajanus* spp. accessions representing two distinct gene pools (primary and secondary): the pre‐domesticated wild species accessions (*C. acutifolius* and *C. cajanifolius*), local landraces (*C. cajan*) and modern elite cultivars or breeding lines (*C. cajan*). *C. cajanifolius* has been considered as the closest wild relative and the progenitor species of cultivated pigeonpea (Kassa *et al*., 2012; Pazhamala *et al*., 2015; Saxena *et al*., 2014). Selected accessions are crossing parents of six mapping populations segregating for economically important traits. This study not only presents the first generation HapMap in *Cajanus* spp. but also reports unique molecular signatures for each accession and large‐scale variations between crossing parents for high‐resolution trait mapping.

## Results

### Selection and features of *Cajanus* spp. accessions

A total of 20 *Cajanus* spp. accessions were selected on the basis of multi‐year/locations trait phenotyping data for development of recombinant inbred line (RIL), introgression line (IL), multiparent advanced generation intercross (MAGIC) and nested association mapping (NAM) populations (Saxena *et al*., 2010; Varshney *et al*., 2010). For instance, ICP 8863 has been found to be resistant to FW, whereas ICP 7035, HPL 24 and ICPB 2049 were resistant to SMD. Two accessions namely, ICPL 99050 and ICPL 20097 were resistant to both FW and SMD. These *Cajanus* spp. accessions differ for a number of agronomic traits (Table S1). Interestingly, these accessions represent all maturity groups starting from super‐early maturity (flowering in <50 days; MN 1), extra‐short maturity (flowering in <70 days; ICPL 85010 and ICPL 88039), short maturity (flowering in <80 days; ICPL 87), short medium (flowering in <110 days; ICP 8863) and medium (flowering in <140 days, ICP 7035, ICP 5529) (Vales *et al*., 2012). Two landraces ICP 7426 and ICP 14209 produce high number of pods per plant whereas, HPL 24 is known as a high seed protein containing line.

### Large‐scale data generation and alignment

Illumina paired‐end sequencing technology was used to sequence a panel of 20 *Cajanus* spp. accessions on the MiSeq platform. As a result, a total of 157 Gb raw data have been generated with 791.77 million reads of read length from 150 to 250 bp (Table 1). Paired‐end reads were mapped onto the reference genome (Varshney *et al*., 2012). Across all 20 accessions, 731.28 million reads were mapped onto the reference genome. Around 93% of total reads from 18 cultivated species accessions and 79% from two wild species accessions were mapped onto the reference genome. These variations in mapping may be due to divergence between parental accessions and the incompleteness of the reference genome assembly. Of these 731.28 million mapped reads, 469.18 million reads were mapped uniquely onto the reference genome, while rest of the reads were mapped to the multiple locations in the genome. Remaining 60.49 million reads (7.6% of total reads) could not be mapped onto the reference genome (Table 1, Figure S1). The effective mapping depth ranged from 8X (MN 1) to 16.5X (ICP 14486) with an average of 12X per *Cajanus* spp. accession. The genome coverage for each *Cajanus* spp. accession against the reference genome varied from 75% to 91% with an average of 89% in the cultivated spp. accessions and 82% in wild species accessions (Table 1).

**Table 1:** Summary of re‐sequencing data generated and genome coverage in 20 *Cajanus* spp. accessions

Genotype	Total reads (million)	Mapped reads		Uniquely mapped reads		Genome coverage (%)	Depth (X)
		Total (million)	%	Total (million)	%		
HPL 24	48.26	45.92	95.16	29.50	61.13	89.19	11.32
ICPB 2049	40.18	37.34	92.94	25.16	62.62	89.10	14.88
ICPL 20097	31.57	28.99	91.84	18.91	59.91	88.66	11.51
ICPL 85010	34.84	32.49	93.25	21.19	60.83	88.50	12.92
ICPL 85063	40.14	37.46	93.31	25.30	63.03	89.31	14.87
ICPL 87	39.36	37.38	94.96	24.50	62.25	88.55	9.20
ICPL 88039	25.91	24.17	93.29	16.29	62.88	86.77	9.59
ICPL 99050	41.41	37.95	91.64	23.73	57.31	89.48	15.12
MN 1	34.03	32.52	95.56	22.37	65.75	87.88	7.82
ICP 11605	38.86	35.78	92.06	22.74	58.51	88.72	14.03
ICP 14209	35.04	32.50	92.74	20.72	59.13	88.52	12.83
ICP 14486	72.22	68.63	95.03	41.74	57.79	90.69	16.46
ICP 28	32.11	29.94	93.24	20.31	63.24	88.26	11.92
ICP 5529	50.10	46.89	93.60	28.04	55.96	89.67	11.36
ICP 7035	36.42	34.52	94.78	23.34	64.10	88.19	8.50
ICP 7263	34.36	32.58	94.82	21.35	62.13	87.94	8.02
ICP 7426	36.31	33.68	92.75	20.53	56.54	88.39	13.38
ICP 8863	43.02	41.04	95.41	25.04	58.20	88.96	9.97
ICPW 12	37.29	24.00	64.37	14.65	39.28	74.63	9.68
ICPW 29	40.34	37.49	92.94	23.76	58.90	89.61	15.16
Total	791.77	731.28		469.18			

### Genome‐wide variations across *Cajanus* spp. accessions

**Figure 1:**
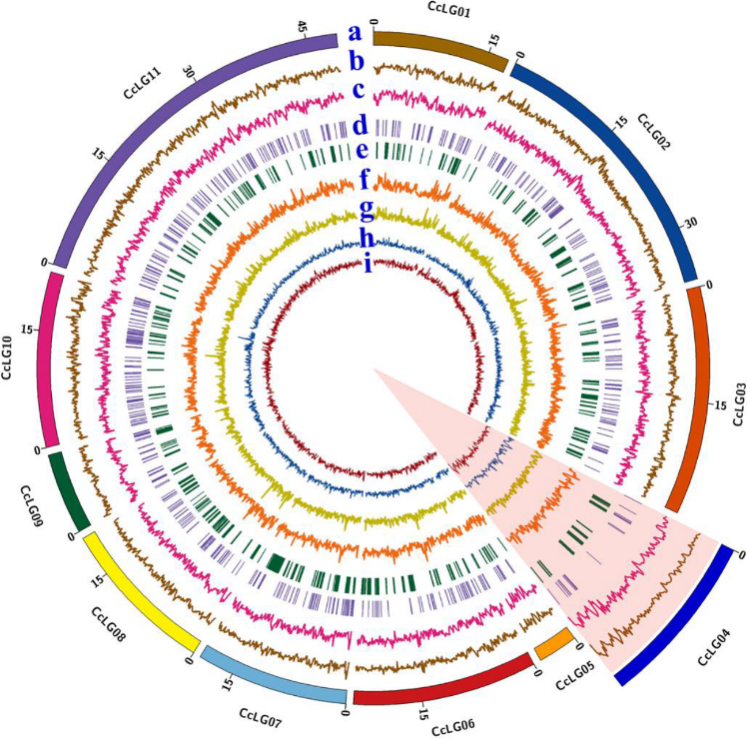
Distribution of genome‐wide variations identified across *Cajanus* spp. accessions against reference genome (the zoom portion of CcLG04 has shown the highest variation densities identified). Different circles (“a” to “i”) represent as following: a: outer most circle represent 11 pseudomolecules, b: SNP density, c: lnDel density, d: presence and absence variations (PAVs), e: copy number variations (CNVs), f: synonymous SNP substitutions density, g: nonsynonymous SNP substitutions density, h: intronic SNP density, i: intergenic SNP density; in the 11 *Cajanus* pseudomolecule.

Genome‐wide SNPs and InDels were identified across *Cajanus* spp. accessions (Figure 1). Comprehensive data analysis identified a total of 5 465 676 variations including 4 686 422 SNPs and 779 254 InDels (373 038 insertions and 406 216 deletions) ranged from 1 to 48 bp in length (Figures S2 and S3). In terms of the assembled genome around 47.5% SNPs, 51.1% insertions and 51.3% deletions were present in 11 pseudomolecules (CcLG01 to CcLG11). Rest of the variations were present on unanchored scaffolds (CcLG0) in draft genome (Table S2). The variations across individual pseudomolecules in *Cajanus* spp. accessions were ranged from 47 453 (CcLG05) to 509 422 (CcLG11). SNPs identified in *Cajanus* spp. accessions were classified into two broad categories, homozygous and heterozygous SNPs based on the presence of one or more than one allele at the same position against the reference genome. The per cent heterozygosity was ranged from 14% in ICPW 12 to 67% in ICPW 29 (Figure S4, Table S3).

SNPs and InDels densities were calculated per 100 kb along each pseudomolecule. The average density of variations across the whole genome was 879.3 SNPs, 82.7 deletions and 75.8 insertions (Figure 1). Densities of SNPs/InDels varied across pseudomolecules. Highest number of SNPs (976), deletions (97) and insertions (91) per 100 kb were observed on CcLG04. The lowest number of SNPs per 100 kb were found on CcLG05 (770), whereas lowest number of deletions (75) and insertions (69) per 100 kb were present on CcLG10 (Table S2). In terms of individual accessions, maximum variations (SNPs and InDels) were identified in wild species accessions (ICPW 12: 3 868 179; ICPW 29: 1 101 494) in comparison with the reference genome. The lowest numbers of variations were present in ICP 8863 (237 170) (Tables 2, S4 and S5).

**Table 2:** Distribution and classification of SNPs in each *Cajanus* spp. accession

Genotype	Total SNPs	Intergenic	Intronic	Exonic						Unknown
				Synonymous	Nonsynonymous					
				Silent	Missense				Nonsense	
					Start lost	Stop lost	Nonsynonymous start	Nonsynonymous coding	Stop gain	
HPL 24	346 096	280 639	42 294	7131	16	64	1	9179	265	6507
ICPB 2049	344 666	289 482	30 652	6121	18	76	2	8862	274	9179
ICPL 20097	199 761	165 235	18 201	4409	14	65	2	6252	200	5383
ICPL 85010	349 778	291 512	32 827	6608	13	80	2	9747	276	8713
ICPL 85063	304 980	255 181	27 054	5867	16	69	3	8611	253	7926
ICPL 87	258 560	213 999	27 442	4932	8	54	2	6907	213	5003
ICPL 88039	287 399	238 046	27 456	5996	18	67	3	8502	239	7072
ICPL 99050	360 154	303 116	32 750	6247	18	81	3	8975	266	8698
MN 1	228 688	189 834	23 834	4065	6	44	2	5926	172	4805
ICP 11605	400 435	335 043	37 427	7393	16	88	2	10 638	295	9533
ICP 14209	338 975	283 217	31 414	6275	17	79	3	9066	284	8620
ICP 14486	404 003	336 672	40 461	7530	18	87	1	10 847	296	8091
ICP 28	335 735	282 033	29 900	6138	17	85	3	8915	253	8391
ICP 5529	258 008	212 661	27 312	5 183	10	58	1	7323	231	5229
ICP 7035	265 762	218 935	28 709	5118	11	50	1	7237	210	5491
ICP 7263	220 759	182 328	23 575	4239	6	34	1	6046	170	4360
ICP 7426	360 166	298 995	33 172	7535	21	99	3	11 070	287	8984
ICP 8863	195 546	161 342	19 415	4331	10	55	2	5946	195	4250
ICPW 12	3 357 515	2 559 376	558 482	92 976	196	386	29	99 797	1360	44 913
ICPW 29	917 145	776 862	85 479	14 449	54	167	4	20 006	486	19 638

Additionally, large variations such as copy number variations (CNVs) and presence and absence variations (PAVs) were also identified across *Cajanus* spp. accessions (Figure 1). A total of 2598 CNVs were found in 2399 genes and 970 PAVs were present in 469 genes across 20 *Cajanus* spp. accessions (Tables S6 and S7). The sizes of identified CNVs were ranged from 1 to 43 kb in *Cajanus* spp. accessions. CNVs and PAVs were nonuniformly distributed across pseudomolecules with maximum on CcLG02 (382 CNVs, 161 PAVs) and minimum on CcLG05 (48 CNVs, 11 PAVs). The highest number of CNVs was found in ICPW 12 (1991) and lowest number of CNVs in ICPL 85063 (03). The highest number of PAVs were found in ICP 14486 (100) and lowest number of PAVs in ICP 7426 (20) (Tables S8 and S9).

Identified SNPs and InDels in *Cajanus* spp. accessions were annotated onto the reference genome. Most of the SNPs were located in intergenic regions (83%) followed by intronic regions (10%), exonic regions (4.8%) and 2.2% could not be classified to any category. Exonic SNPs were further classified into synonymous and nonsynonymous SNPs, and the resulting ratio of nonsynonymous to synonymous substitutions was 1.46. Furthermore, the effect of each SNP was also categorized into four classes, viz. modifier, low effect, moderate effect and high effect. About 93% SNPs (presented in intergenic and intronic regions) were classified as modifier. Remaining 4.8% exonic SNPs were classified as low effect (silent‐synonymous substitutions and nonsynonymous start), moderate effect (nonsynonymous coding) and high effect (start lost, stop lost and stop gain) (Tables 2 and S10).

The effects of InDels were categorized separately as insertion and deletion effects (Tables S4 and S5). However, majority of InDels were located in intergenic (76%–86%) and intronic region (11%–21%) according to the parental accessions and considered as modifier. Across parental accessions, only 1%–1.5% InDels were located in exonic regions. The moderate effect insertions were classified as codon insertion and codon change plus codon insertion while deletions were codon deletion and codon change plus codon deletion. These moderate effect InDels were in‐frame 3 bp InDels (a multiple of three) and were found in the range of 131 (MN 1) to 2871 (ICPW 12). High‐effect InDels were classified as start lost, stop lost, stop gain and frame shift. Frame shift mutations caused by out‐of‐frame InDels (not a multiple of three) were found in the range of 269 (ICP 8263) to 2453 (ICPW 12).

Furthermore, repetitive sequences in the form of tandem repeats and interspersed repeats were also identified in each *Cajanus* spp. accession. The transposable elements or interspersed repeats were classified into retrotransposons and DNA transposons. In all the *Cajanus* spp. accessions, the majority of transposable elements were long terminal repeats (LTRs). The highest number of LTRs were found in ICPW 12 (639 419), whereas the lowest number were found in ICP 7263 (57 489) (Table S11).

### Candidate variations for trait mapping

To detect parental polymorphism, pairwise SNPs and InDels were identified between each crossing parental (Table S12). In the case of bi‐parental mapping populations, maximum SNPs were present between crossing parentals of Introgression libraries‐1 (IL‐1) (ICPW 12 vs ICPL 87119: 3 357 515) followed by Introgression libraries‐2 (IL‐2) (ICPW 29 vs ICPL 87119: 917 145), PRIL_A (ICPB 2049 vs ICPL 99050: 307 593) and PRIL_C (ICPL 20097 vs ICPL 8863: 201 986) (Table S13). In the multiparent mapping populations, pairwise comparisons were made in all the possible combinations. For instance, a set of 28 crossing combinations in MAGIC and 10 crossing combinations in NAM were compared. In MAGIC population, maximum number of SNPs and InDels were found between HPL 24 × ICP 14486 (436 293 and 97 757, respectively) and minimum between ICP 5529 × ICP 8863 (235 332 and 61 819, respectively) (Table S14). In the NAM population, pairwise SNPs and InDels were maximum in ICPL 85010 × ICPL 87119 (349 778 and 71 250, respectively) and minimum in ICP 8863 × ICPL 87119 (195 546 and 41 625, respectively) (Table S12).

### Genetic relationships among *Cajanus* spp. accessions

**Figure 2:**
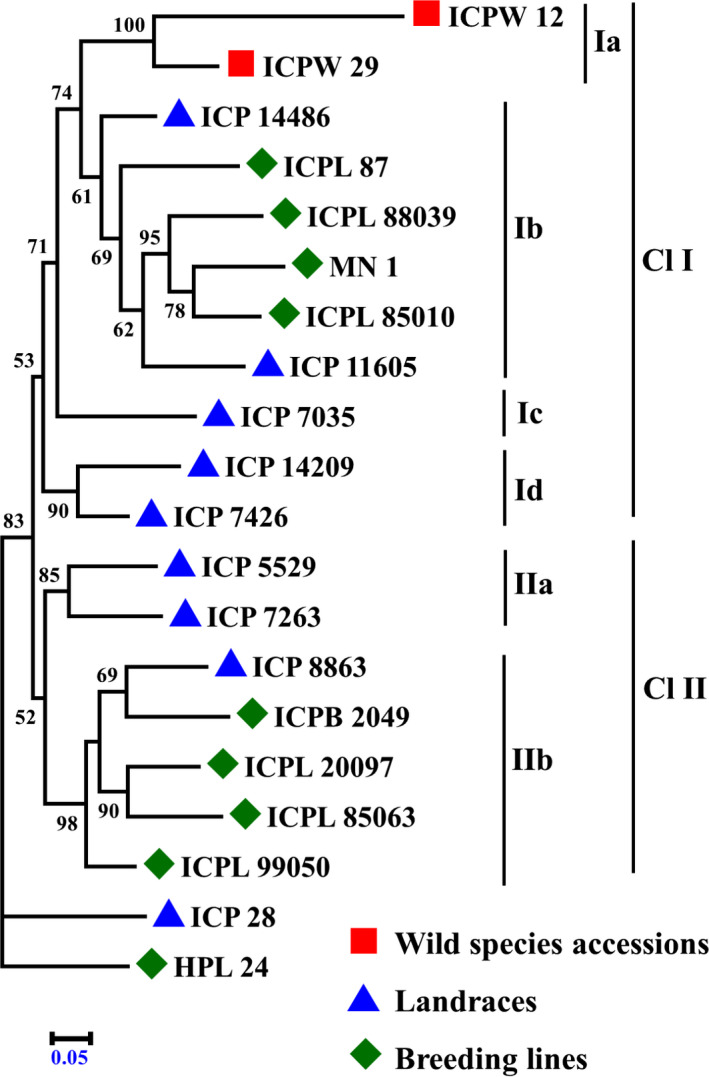
Phylogenetic relationships in 20 *Cajanus* spp. accessions: wild species accessions (square shape in red color), landraces (triangle shape in blue color) and breeding lines (diamond shape in green color).

To explain the genetic divergence among the *Cajanus* spp. accessions, a phylogenetic tree was constructed with the help of SNPhylo program (Figure 2). *Cajanus* spp. accessions ICP 28 and HPL 24 were out‐grouped from rest of the accessions. Remaining accessions were broadly categorized into two main clusters (Cl I and Cl II). Cl I contained 11 accessions representing two wild species accessions, five landraces and four breeding lines, whereas Cl II contained seven accessions representing three landraces and four breeding lines. Under these two main clusters, accessions were grouped further into subclusters. For instance, the cluster Cl I contained four subclusters (Ia, Ib, Ic and Id). Wild species accessions were grouped into a small subclusters Cl Ia, four breeding lines with two landraces were grouped in Cl Ib, and ICP 7035 was placed in subcluster Cl Ic, while the landraces ICPL 14209 and ICPL 7426 were grouped together in a subcluster Cl Id. Furthermore, the cluster Cl II contained two subclusters (IIa and IIb). Landraces ICP 5529 and ICP 7263 were grouped in Cl IIa, while the remaining 4 breeding lines and one landrace were grouped in Cl IIb. To assess divergence among 8 parental accessions of MAGIC population and 11 parental accessions of NAM population, separate dendrograms were also constructed (Figure S5). Pairwise genetic distances between wild species accessions were greater than those between any pairs of the domestic accessions, that is breeding lines and landraces. Diversity between any pair of the wild species accession with domestic pigeonpea accession is greater than those among all possible domestic pigeonpea accessions pairs (Table S15).

### Accession‐specific variations

**Figure 3:**
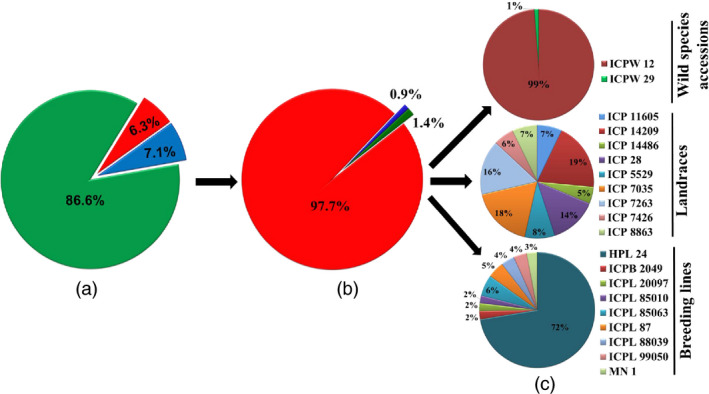
Distribution of accession‐specific or unique variations (SNPs, insertions and deletions) across 20 *Cajanus* accessions: (a) percentage of accession‐specific SNPs, insertions and deletions identified in total unique variations represented as 86.6% accession‐specific SNPs (green color), 6.3% accession‐specific deletions (red color), 7.1% accession‐specific insertions (blue color), (b) percentage of accession‐specific variations identified within three groups represented as 97.7% in wild species accessions group (red color), 0.9% in landraces group (blue color) and 1.4% in breeding lines group (green color), (c) percentage of accession‐specific variations in each accession, individually within a group represented by a specific color (wild species accessions group represented two accessions, landraces and breeding lines groups represented nine accessions in each).

A total of 2 630 904 accession‐specific variations (SNPs and InDels) were found in all *Cajanus* spp. accessions. These accession‐specific variations include 2 278 571 (86.6%) SNPs, 166 243 (6.3%) deletions and 186 090 (7.1%) insertions (Table 3). The proportion of accession‐specific variants ranged from 0.2% to 65.6% of the total variants. The accession ICPW 12 had the highest number of accession‐specific variants 2 537 481 (65.6% of the total variants). However, on the other side, ICPL 85010 had the lowest number of accession‐specific variants 791 (0.2% of the total variants). Among the accession‐specific variants, wild species accession‐specific (97.7%) were more abundant than landrace‐specific (1.4%) and breeding line‐specific (0.9%) variants (Figure 3). The frequency of accession‐specific variants located in exonic regions were ranged from 2.9% in ICPB 2049 to 5.6% in ICP 14486 (Table S16). However, the total number of accession‐specific variants (SNPs and InDels) located in exonic region ranged from 24 (ICPB 2049) to 120 336 variants (ICPW 12). Gene ontology annotation was used to assess possible gene functions targeted by accession‐specific variants for all 20 accessions, and details have been provided in Table S17. In addition to these accession‐specific small variations, some accession‐specific large variations in the form CNVs (2258) and PAVs (288) were also identified (Table 3).

**Table 3:** Accession‐specific small variations (SNPs, deletions and insertions) and large variations (CNVs and PAVs with ≥1 kb) present in *Cajanus* spp. accessions

Genotype	SNPs	Deletions	Insertions	CNVs	PAVs
HPL 24	22 864	1685	2101	0	35
ICPB 2049	647	86	102	4	9
ICPL 20097	636	81	99	3	8
ICPL 85010	601	94	96	13	21
ICPL 85063	1672	233	261	0	3
ICPL 87	1312	174	209	5	18
ICPL 88039	1083	121	194	201	8
ICPL 99050	1074	128	170	7	11
MN 1	829	114	150	2	7
ICP 11605	1378	189	201	12	18
ICP 14209	3840	414	544	19	7
ICP 14486	912	138	138	1	26
ICP 28	2672	360	356	13	1
ICP 5529	1647	229	242	0	20
ICP 7035	3526	420	457	4	13
ICP 7263	3061	359	450	1	7
ICP 7426	1108	124	211	28	9
ICP 8863	1342	194	255	0	12
ICPW 12	2 202 592	158 075	176 814	1943	23
ICPW 29	25 775	3025	3040	2	32
Total	2 278 571	166 243	186 090	2258	288

## Discussion

Pigeonpea productivity just has not suffered from biotic, abiotic stresses, low polymorphism in cultivated gene pool, but also from less genomics and genetic resources in the past (Pazhamala *et al*., 2015; Varshney *et al*., 2010). However, in recent years, NGS technologies were successfully deployed in pigeonpea, and ample genomic resources have been developed such as draft genome assembly (Varshney *et al*., 2012) and transcriptome assemblies (Dubey *et al*., 2011; Kudapa *et al*., 2012). Above‐mentioned advances in pigeonpea genomics have provided opportunities to assess genome‐wide variations using WGRS approach. WGRS in the present study identified more than 4.5 million SNPs, 779 254 InDels along with 2598 CNVs and 970 PAVs distributed throughout the *Cajanus* genome. Prior to this study, only few thousands of SNPs were reported by aligning transcriptome reads from 12 pigeonpea genotypes onto the pigeonpea reference genome (Dubey *et al*., 2011; Varshney *et al*., 2012). WGRS efforts have been undertaken to extend the repertoire of polymorphisms in a number of crop species such as rice (Huang *et al*., 2012; Xu *et al*., 2012), maize (Hufford *et al*., 2012), sorghum (Mace *et al*., 2013), chickpea (Varshney *et al*., 2013), *Medicago* (Stanton‐Geddes *et al*., 2013), soybean (Lam *et al*., 2010; Zhou *et al*., 2015), tomato (Lin *et al*., 2014), etc. Notably in *Medicago*, more than 6 million SNPs were identified through re‐sequencing of 236 diverse accessions at ~8X coverage (Stanton‐Geddes *et al*., 2013). In continuation of this work, 384 inbred lines are being re‐sequenced under *Medicago* HapMap project using Illumina NGS technology to discover the SNPs, InDels and CNVs at 5X to 20X coverage (http://www.medicagohapmap.org). In the case of chickpea, a total of 82 489 genome‐wide SNPs (including 38 511 and 43 978 from *desi* and *kabuli* genomes, respectively) were identified from 93 *Cicer* accessions through integrated reference genome‐ and *de novo*‐based GBS assays (Bajaj *et al*., 2015). To assess genome‐wide variations and their faster deployment in breeding programs, it is highly essential to select high priority accessions. In this study, a set of 20 *Cajanus* spp. accessions has been selected which are of high priority in pigeonpea breeding program. Similarly, three male‐sterile and three restorer lines in rice (Subbaiyan *et al*., 2012), two parents of a bi‐parental population in soybean (Li *et al*., 2014) and eight parents of MAGIC population in tomato (Causse *et al*., 2013) were subjected to WGRS.

Sequencing data from 20 *Cajanus* spp. accessions were mapped onto the reference genome. The average mapping rate in *Cajanus* spp. accessions was 92.4%, which is comparable to other re‐sequencing efforts in different crop species (Causse *et al*., 2013; Mace *et al*., 2013). Mapping % of the sequence reads in wild species accessions (79%) was low as compared to cultivated species accessions (93%). The mapping % of sequence reads generated from wild species accessions onto the cultivated reference genomes has also been found to be low in rice (81%; Xu *et al*., 2012), sorghum (89%; Mace *et al*., 2013), etc. This seems to be a factor of complex genome present in wild species as compared to cultivated species accessions. However, total genome coverage in wild *Cajanus* spp. accessions (82%) was comparable to cultivated species accessions (89%). Around 7.6% of the total reads generated in *Cajanus* spp. accessions could not be mapped onto the reference genome. This rate is lower than unmapped reads in rice (9.5%, Arai‐Kichise *et al*., 2011; 10%, Xu *et al*., 2012; 15%, Subbaiyan *et al*., 2012) and comparatively higher than 5% of unmapped reads in tomato (Causse *et al*., 2013).

To assess genomic diversity in *Cajanus* spp., different marker systems have been developed such as random amplified polymorphic DNA, amplified fragment length polymorphism, DArT, SFP, SSRs, genic SSR and SNPs (Pazhamala *et al*., 2015). Above‐mentioned markers could not exceed more than few thousands in numbers. Moreover, less genetic diversity present in cultivated pool further hampered their use in assessing genome‐wide diversity, linkage mapping and trait association analysis at higher confidence. However, a few thousand SNPs have been used for the development of genetic map and quantitative trait locus analysis (Kumawat *et al*., 2012; Saxena *et al*., 2012) as well as for genetic diversity analysis (Kassa *et al*., 2012; Saxena *et al*., 2014) in *Cajanus* species. The present study was sought to develop millions of markers so that high‐resolution trait mapping can be undertaken. WGRS of *Cajanus* spp. accessions have provided ~4.6 million SNPs. However, the number of SNPs in landraces and breeding lines were comparatively lesser than wild species accessions. Previous marker‐based genotyping on *Cajanus* spp. accessions also suggested the lower diversity in cultivated pool as compared to wild species (Kassa *et al*., 2012; Saxena *et al*., 2014). As a next step to perform high‐density genotyping of mapping populations, one high‐density SNP array has been planned to develop with the most informative SNPs (high PIC value and uniform distribution in genome) identified in this study. High‐density SNP arrays have been developed and found suitable for genotyping in rice (McNally *et al*., 2009), maize (Ganal *et al*., 2011), soybean (Song *et al*., 2013), etc.

Another class of small variations, that is InDels (0.7 million) were detected in *Cajanus* spp. accessions. The number of InDels was decreased as the length of InDel increased. InDels with 1 bp length variation were found maximum (52%) followed by 2–4 bp (33%), 5–9 bp (10%) and remaining 10–48 bp length (5%). Similar frequency (>50%) of mononucleotide InDels have been reported in rice (Arai‐Kichise *et al*., 2011; Subbaiyan *et al*., 2012) and sorghum (Mace *et al*., 2013). InDels with length ≥5 bp could easily show length polymorphism similar to microsatellite markers on agarose gel (Arai‐Kichise *et al*., 2011) and can be converted as breeder‐friendly InDel markers (Tao *et al*., 2014). Informative InDels of any length from 1 to 48 bp can be used for InDel array development (Salathia *et al*., 2007) or can integrate them (InDels) into SNP array to increase their power in genotyping of several mapping populations (Unterseer *et al*., 2014).

In the present study, we have identified 5.5 million genome‐wide variations across parental accessions representing elite breeding lines, landraces and wild species accessions, which are fractionally higher from 1.1, 4.3 and 5.1 million variations identified in parental lines in soybean (Li *et al*., 2014), tomato (Causse *et al*., 2013) and rice (Subbaiyan *et al*., 2012), respectively. Annotation of identified variations in 20 accessions showed <5% variations in coding regions. This proportion is similar to the ratio of 4.5% of SNP and 1.5% of InDel identified in sorghum (Mace *et al*., 2013). Among coding regions, the proportion of nonsynonymous SNPs is higher than that of synonymous SNPs. The ratio of nonsynonymous to synonymous substitutions in *Cajanus* spp. accessions was found within the range of earlier reports in other crop species viz. soybean: 1.37 (1.36 in wild and 1.38 in cultivated; Lam *et al*., 2010), tomato: 1.41 (1.34 and 1.48 in cherry tomato and cultivated tomato lines, Causse *et al*., 2013) and rice: 1.29 (Xu *et al*., 2012). Additionally, CNVs and PAVs have been identified in *Cajanus* spp. accessions. Interestingly, results suggested that the frequency of gene lost events is higher in accessions which are under cultivation (landraces and breeding lines) as compared to the wild species accession.

## Conclusion

Variants discovered from re‐sequencing of 20 *Cajanus* spp. accessions offer information on sampled loci across the pigeonpea genome harboring high diversity and unique accession signatures. These polymorphic sites will be useful for developing high‐density SNP arrays, genotyping of several mapping populations to construct genetic maps and identify the genomic regions responsible for agronomic important traits. Unique accession signatures will be useful in varietal identification or assessment of adoption of varieties in different geographies. This study also re‐emphasized that the cultivated pigeonpea genepool has a narrow genetic base and new populations such as IL, MAGIC, NAM, etc. must be used to re‐introduce adaptive diversity lost through domestication and human selection in breeding. To use new genetic combinations, it is highly recommended to have high‐density genotyping to track the superior haplotypes and avoid linkage drag in breeding programs aimed for pigeonpea improvement.

## Experimental procedures

### Plant materials

A set of 20 *Cajanus* spp. accessions including two wild species accessions, nine breeding lines and nine landraces were used in this study. These accessions are crossing parents of six mapping populations, including four bi‐parental and two multiparent mapping populations. Among these, ICPW 12 (*C. acutifolius*) and ICPW 29 (*C. cajanifolius*) are wild *Cajanus* spp. accessions and used for the development of two AB populations, namely IL‐1 and IL‐2, respectively. Accession ICPL 87119 from *C. cajan* was used as a common crossing parent in both the above‐mentioned ILs. ICPL 87119 is a leading variety which released as ‘Asha’ in 1992 in India; subsequently, it was used in developing pigeonpea draft genome (Varshney *et al*., 2012). Four accessions (ICPB 2049, ICPL 99050, ICPL 20097 and ICP 8863) were parents of two pigeonpea recombinant inbred lines: PRIL_A (ICPB 2049 × ICPL 99050) and PRIL_C (ICPL 20097 × ICP 8863). Eight accessions (ICP 5529, HPL 24, ICP 7035, ICP 8863, ICP 14486, ICP 11605, ICP 7426 and ICP 14209) representing breeding lines and landraces are being used in developing MAGIC population. 10 accessions (HPL 24, ICPL 85010, ICPL 85063, ICPL 87, ICPL 88039, MN 1, ICP 28, ICP 7035, ICP 7263 and ICP 8863) as founder parents and ICPL 87119 as a nested parent are being used in developing NAM population. Details on each accession have been provided in Table S1.

### Library preparation and sequencing

Genomic DNA was extracted from young leaves of individual plants of each accession, using a NucleoSpin Plant II kit (Macherey‐Nagel, Düren, Germany). DNA quality was checked on 0.8% agarose gel and DNA quantity assessed on Qubit^®^ 2.0 Fluorometer using the dsDNA BR Assay kit (Life Technologies, Thermo Fisher Scientific Corporation, Waltham, MA). Indexed DNA libraries were prepared following Illumina paired‐end DNA sample prep protocol (Part # 15026486 Rev.C, July 2012) with minor modifications. Initially, 2 μg of genomic DNA from each sample was sonicated by Bioruptor^®^ NGS (Diogenode, Liege, Belgium) to get a target insert size of 400–500 bp. After end‐repairing and indexed adapter ligation, size selection of 600 bp DNA fragments was performed on E‐Gel^®^ SizeSelect^™^ 2% agarose precast gels (Invitrogen, Life Technologies, Thermo Fisher Scientific Corporation, Waltham, MA). To enrich DNA fragments having adapters on both ends, PCRs were performed using Illumina adapter compatible PCR primers. The size distribution of amplified DNA libraries was checked on Agilent 2100 Bioanalyzer (Agilent Technologies, Palo Alto, CA). DNA libraries were sequenced on the MiSeq platform (Illumina Inc., San Diego, CA) with MiSeq reagent kit v2 (300‐ or 500‐cycles) to generate 150 or 250 bp paired‐end reads.

### Sequence alignment and variant identification

To reduce sequencing errors, paired‐end sequencing reads were trimmed and filtered with sickle version 1.200 (https://github.com/najoshi/sickle). Initially, duplicate reads were removed, further low‐quality reads (having phred score <30) and sequences shorter than 100 nucleotides, or containing ‘N’, were removed using in‐house QC pipeline NGS‐QCbox (Katta *et al*., 2015). After cleaning steps, filtered reads were mapped onto the reference genome with bowtie2 v2.2.4 (Langmead and Salzberg, 2012) using default options. Reads mapped on more than one position or not mapped were filtered to define uniquely mapped reads and unmapped reads. Reads having unique alignment onto the reference genome were retained in the BAM files. BAM files were further processed for variant (SNP and InDel) calling using Genome Analysis Toolkit suite with a minimum depth coverage of five reads per individual accession (McKenna *et al*., 2010). Using an in‐house perl script, the distribution of identified variants was analysed along the entire genome using a contiguous window of 100 kb. Additionally, identified SNPs were classified into homozygous and heterozygous (reads aligned at a position contained reference as well as alternate bases) SNPs, on the basis of mismatch frequencies. InDels were identified within the size range of 1–48 bp. Accession‐specific variants (SNPs and InDels) were reported only if the variant call was present in a particular accession and reference allele was present in remaining accessions. For identification of CNVs, CNVnator tool was used with an e‐value of 1e‐05 (Abyzov *et al*., 2011). Raw reads from Asha (ICPL 87119) genotype were aligned to draft assembly (Asha) for detecting the false positives in CNVs. Identified CNVs present in genes with length ≥1 kb were then reported. The frequencies of variants (SNPs and InDels) and CNVs were then projected using Circos (Krzywinski *et al*., 2009) across the categorized genotypes.

### Variant annotation and diversity analysis

Identified SNPs and InDels based on their genomic locations were annotated as intergenic, intronic and exonic using SnpEff (Cingolani *et al*., 2012). The variants were further categorized into synonymous, nonsynonymous, start codon loss, stop codon gain, frame shifts, etc. The effects of variants were classified on the basis of their impacts as high, moderate, low and modifier. Generic feature format files having information on positions of variants were constructed by aligning the sequences against the reference genome. Accession‐specific variants (SNPs and InDels) present in exonic region for each accession were functionally annotated using UniProtKB database, and GO terms were assigned accordingly (Huntley *et al*., 2014). Further, the impacts of accession‐specific variants in various biological pathways were examined using The Biological Networks Gene Ontology tool (Maere *et al*., 2005).

The phylogenetic tree was constructed using DNAML programs in the PHYLIP package and 1000 bootstraps with other default parameters of SNPhylo program (Lee *et al*., 2014). The software mega4 was used for visualizing the phylogenetic tree (Tamura *et al*., 2007).

## Supporting information


**Figure S1** Classification of the sequencing reads generated through re‐sequencing of 20 *Cajanus* spp. accessions and mapped onto the reference genome.

**Figure S2** Genome‐wide distribution of InDels with varying length.

**Figure S3** Distribution of insertions (blue color) and deletions (red color) identified in 20 *Cajanus* spp. accessions.

**Figure S4** Distribution of homozygous (green color) and heterozygous (red color) SNPs identified between each of the 20 *Cajanus* spp. accessions and reference genome.

**Figure S5** Phylogenetic relationships among: (a) 8 MAGIC parental lines as landraces (triangles shape in blue color) and breeding lines (diamonds shape in green color) (b) 10 NAM founder and 1 nested parental line represented by landraces (triangles shape in blue color), breeding lines (diamonds shape in green color) and nested parent (circle shape in chocolate color).

**Table S1** Details of *Cajanus* spp. accessions included in the present study.

**Table S2** Distribution of genome‐wide variations in different pseudomolecules.

**Table S3** Heterozygosity of SNPs in *Cajanus* genome.

**Table S4** Distribution of insertions and their effects in *Cajanus* spp. accessions.

**Table S5** Distribution of deletions and their effects in *Cajanus* spp. accessions.

**Table S6** Location of genes deleted in *Cajanus* spp. accessions.

**Table S7** Location of genes duplicated in *Cajanus* spp. accessions.

**Table S8** Summary of large deletions (>1 kb) in *Cajanus* spp. accessions.

**Table S9** Summary of large duplications (>1 kb) in *Cajanus* spp. accessions.

**Table S10** Distribution of homozygous SNPs and their effects.

**Table S11** Distribution of tandem and interspersed repeats in *Cajanus* spp. accessions.

**Table S12** Pairwise SNPs (below diagonal) and InDels (above diagonal) among *Cajanus* spp. accessions.

**Table S13** Pairwise SNPs and InDels between parental lines of biparental populations.

**Table S14** Pairwise SNPs (below diagonal) and InDels (above diagonal) between MAGIC parental lines.

**Table S15** Pairwise genetic distances among 20 selected *Cajanus* spp. accessions.

**Table S16** Distribution of accession‐specific variants and their effects.

**Table S17** Accession‐specific variants and their effects on coding sequences and Gene Ontology (GO).
