# Supplementary figures and images for: First‐generation HapMap in Cajanus spp. reveals untapped variations in parental lines of mapping populations
Source: Plant Biotechnol J. 2016 Jan 29;14(8):1673–81. doi: 10.1111/pbi.12528 (PMC5066660; doi:10.1111/pbi.12528)

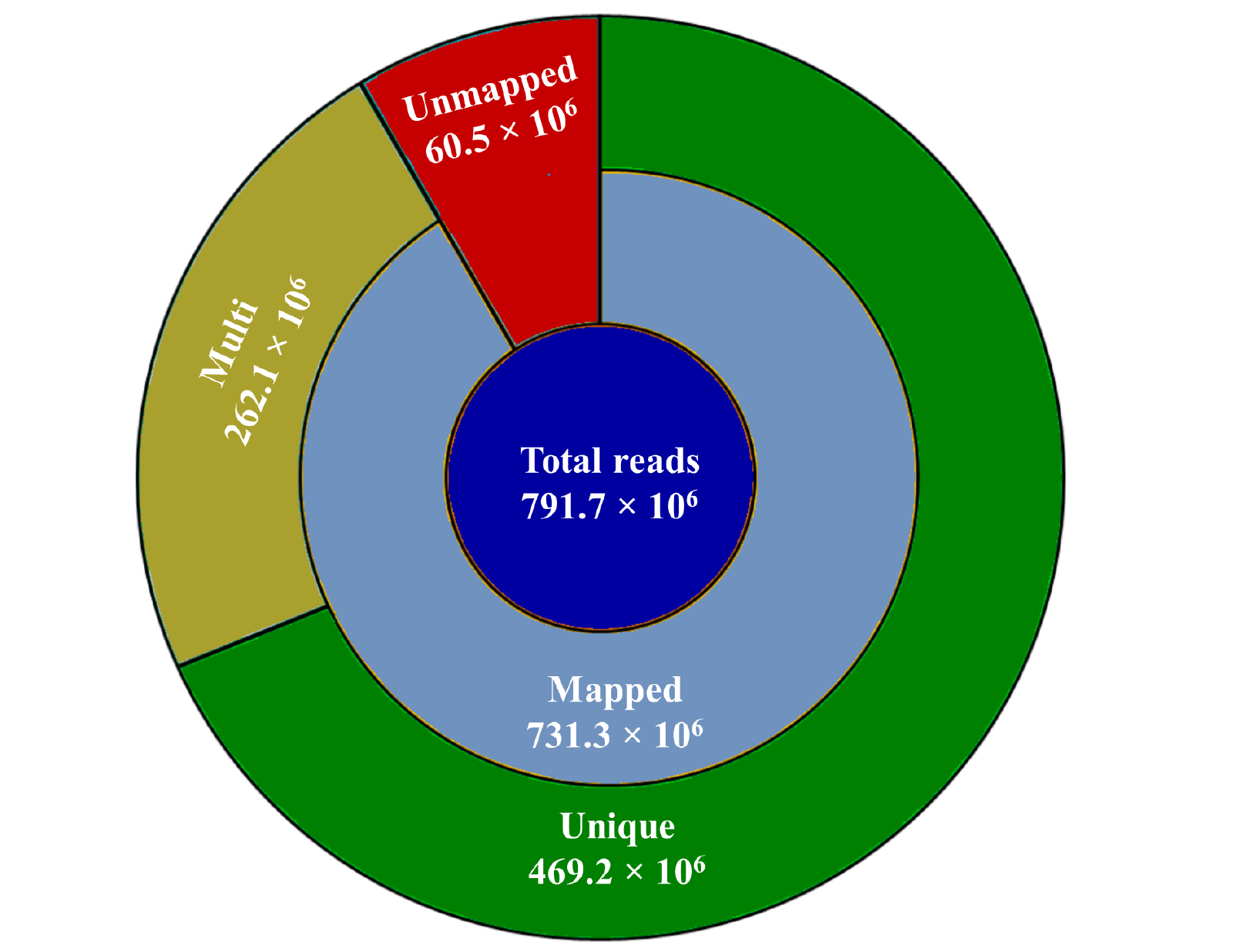

Supplement: Supplementary file 1 — Figure S1 Classification of the sequencing reads generated through re‐sequencing of 20 Cajanus spp. accessions and mapped onto the reference genome. [file PBI-14-1673-s021.tif]

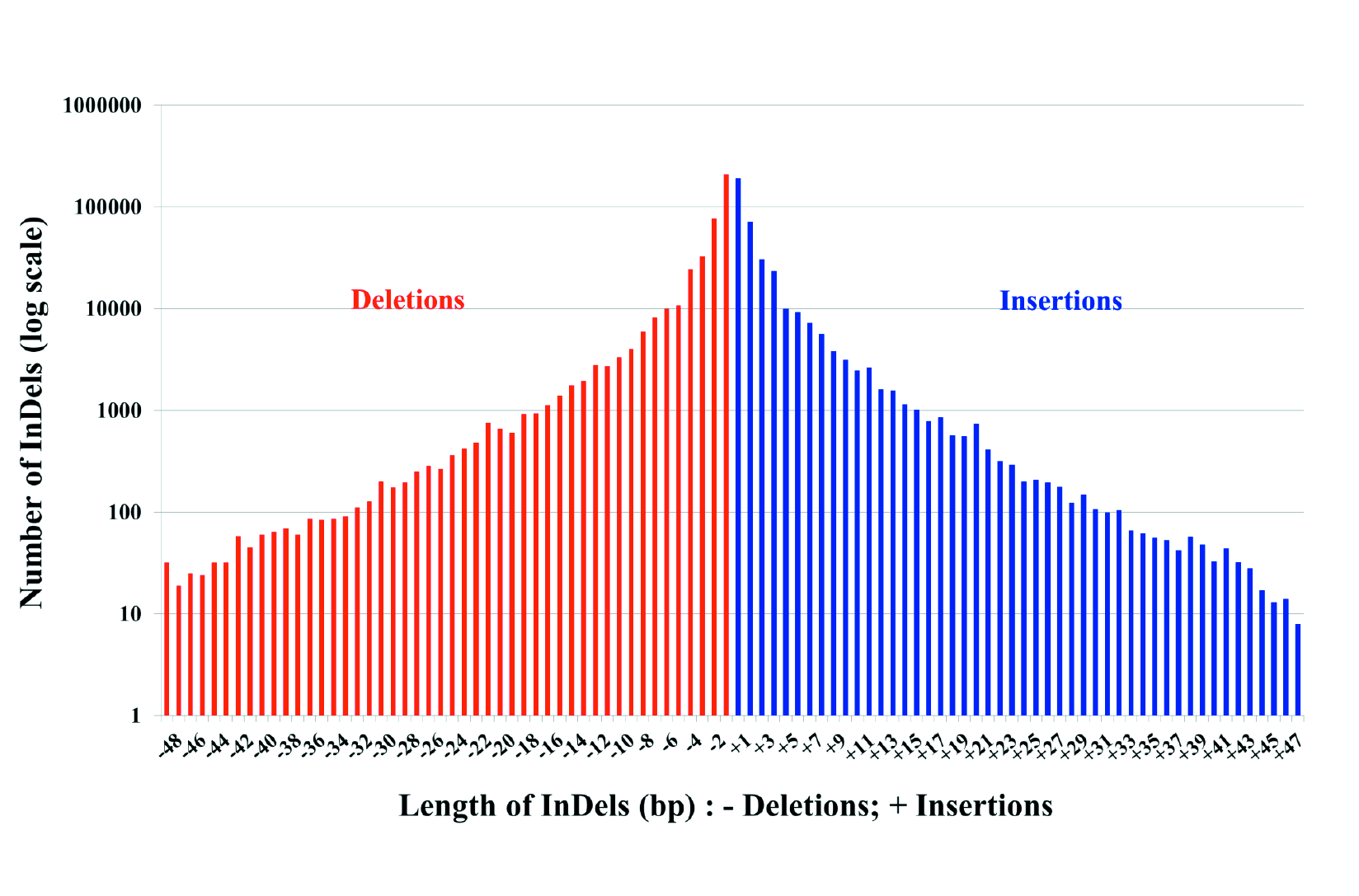

Supplement: Supplementary file 2 — Figure S2 Genome‐wide distribution of InDels with varying length. [file PBI-14-1673-s022.tif]

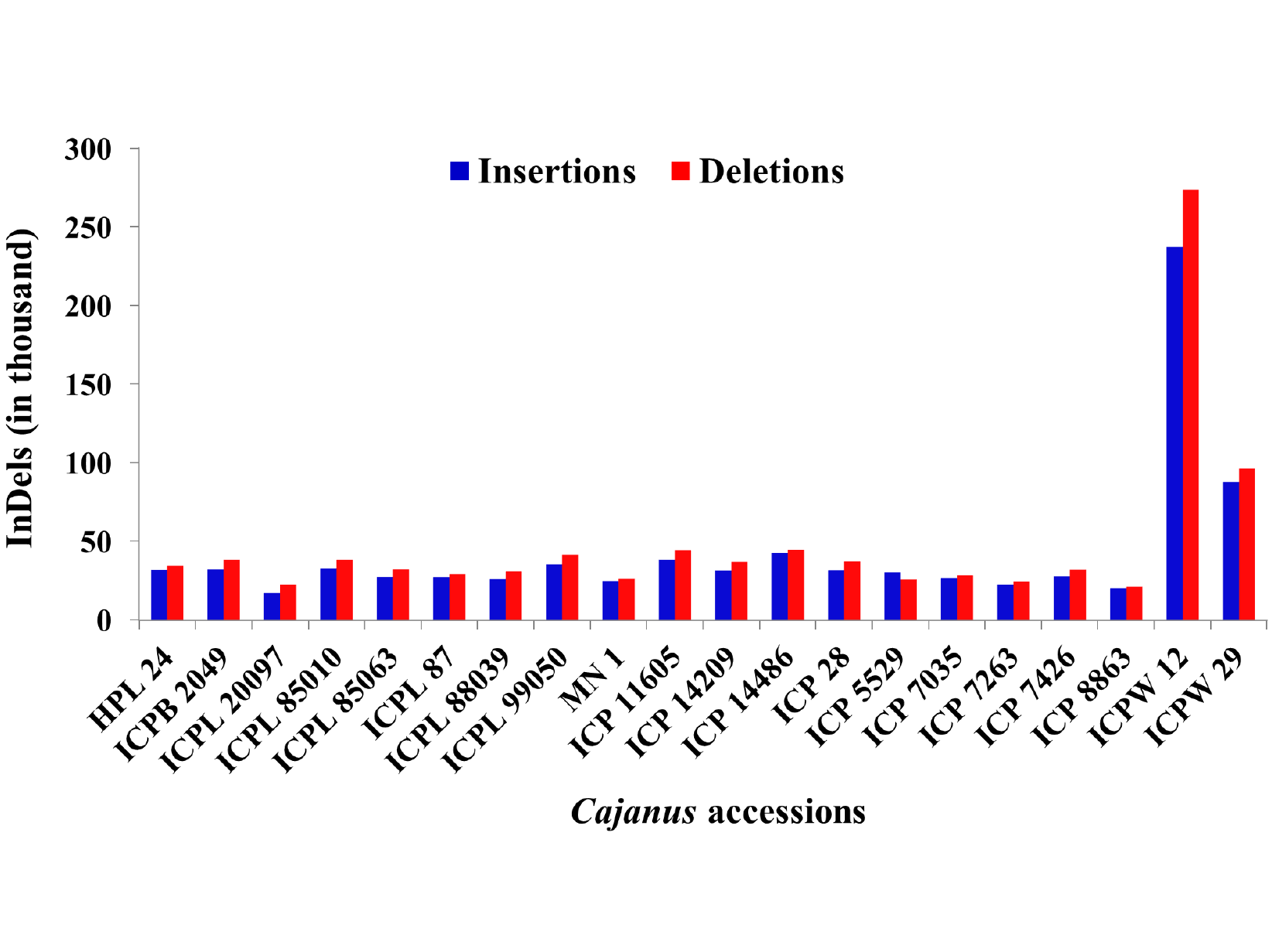

Supplement: Supplementary file 3 — Figure S3 Distribution of insertions (blue color) and deletions (red color) identified in 20 Cajanus spp. accessions. [file PBI-14-1673-s020.tif]

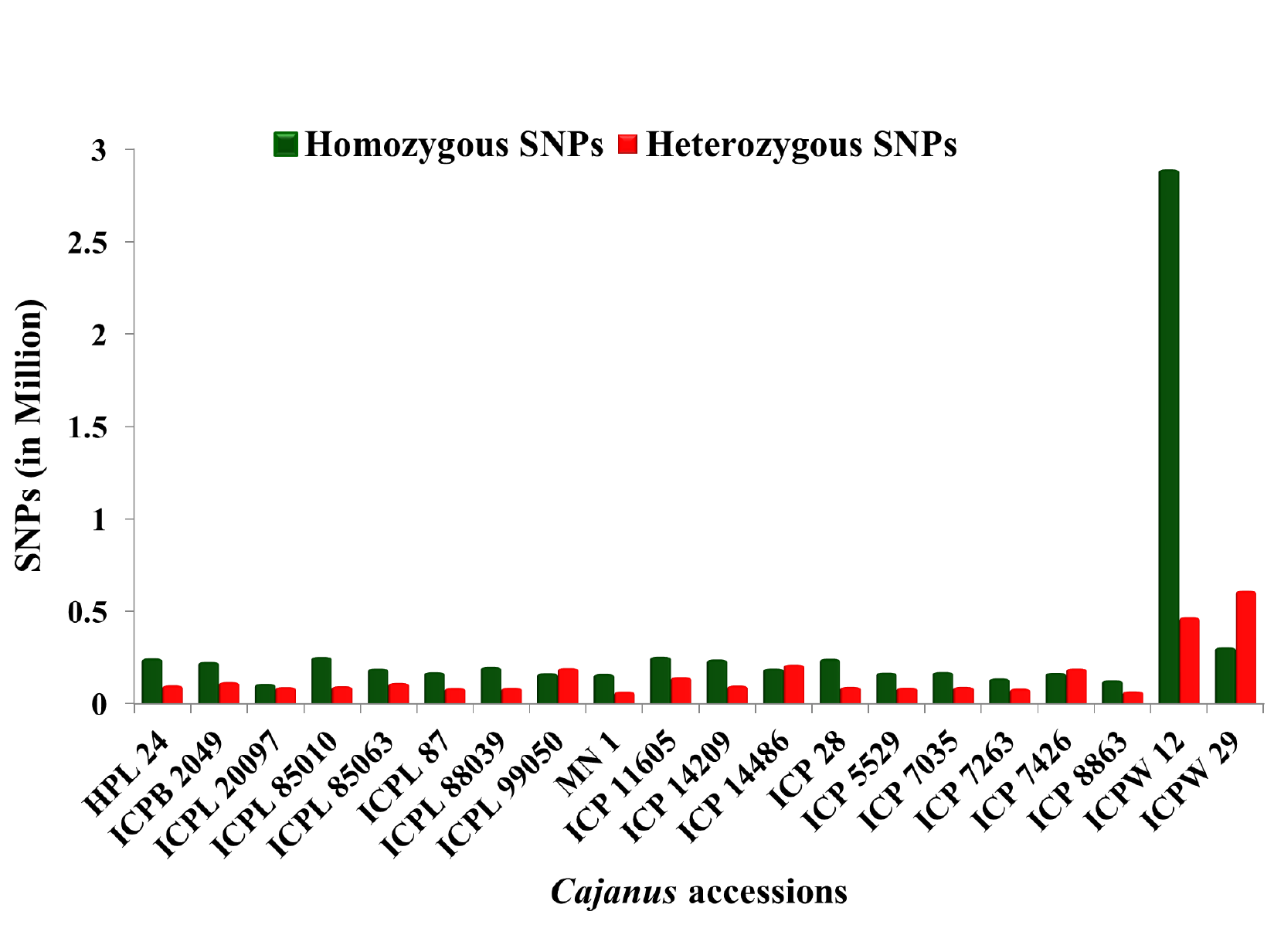

Supplement: Supplementary file 4 — Figure S4 Distribution of homozygous (green color) and heterozygous (red color) SNPs identified between each of the 20 Cajanus spp. accessions and reference genome. [file PBI-14-1673-s019.tif]

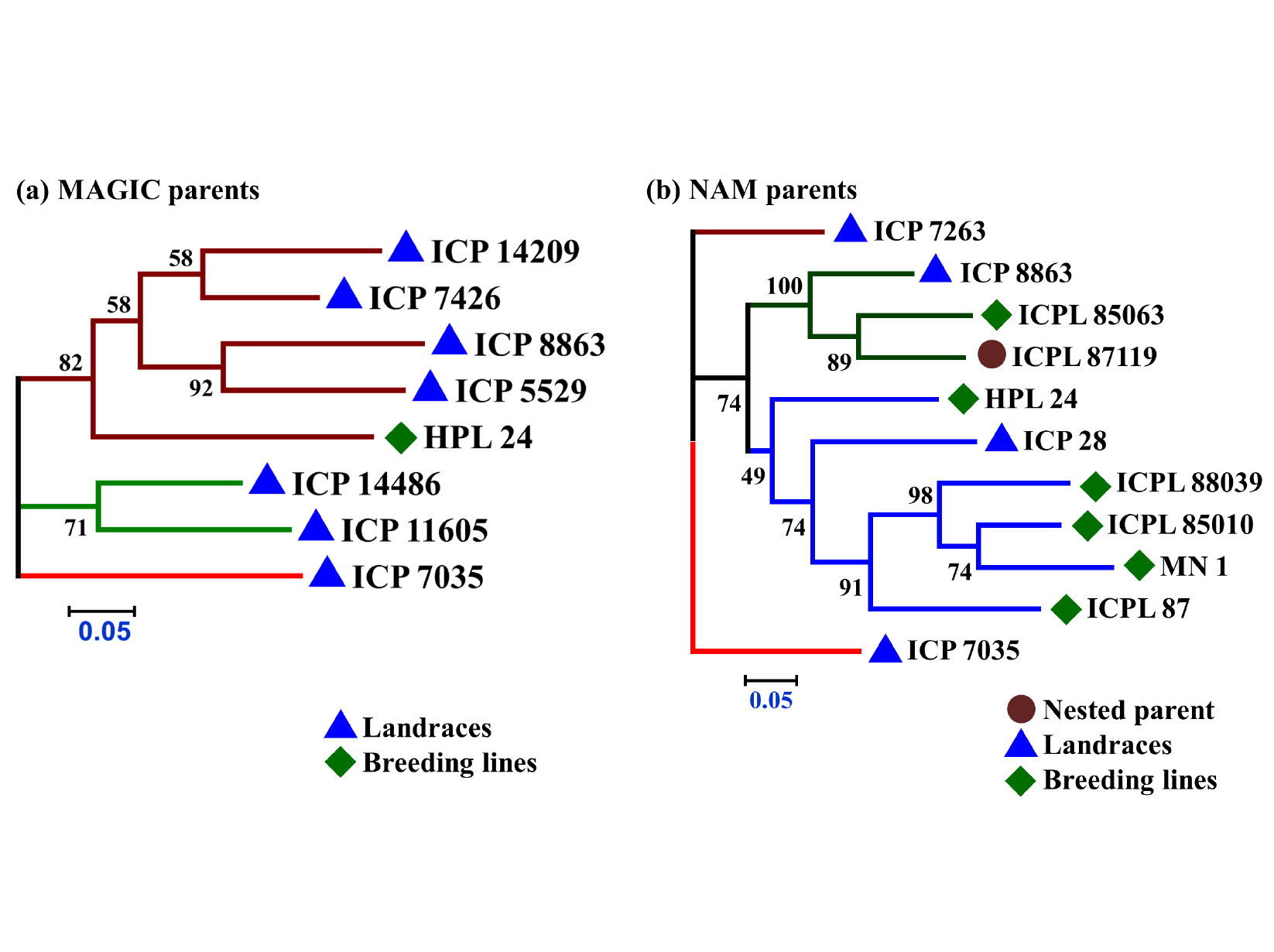

Supplement: Supplementary file 5 — Figure S5 Phylogenetic relationships among: (a) 8 MAGIC parental lines as landraces (triangles shape in blue color) and breeding lines (diamonds shape in green color) (b) 10 NAM founder and 1 nested parental line represented by landraces (triangles shape in blue color), breeding lines (diamonds shape in green color) and nested parent (circle shape in chocolate color). [file PBI-14-1673-s018.tif]
